# Dr. J. W. Ash’s Case of Latent Typhoid Fever

**Published:** 1842-04-16

**Authors:** J. W. Ash

**Affiliations:** Delaware Co., Pa.


### *Case of latent Typhoid Fever terminating fatally.*

By J. W. Ash, M. D., of Delaware Co., Pa.

[Within some months past a local epidemic of typhoid fever has prevailed in a portion of Delaware county, about seven miles west of the city of Philadelphia. The epidemic has been limited to an irregular circle of about five miles in diameter; beyond these limits the disease is much less common than usual; in the city of Philadelphia it has been quite rare during the past winter. The disease received various appellations—congestive, nervous, typhus fever, &c. given capriciously enough. It is very clear, however, that it was essentially the same affection; and, as is usual in epidemics, it differed in some respects from the sporadic form of the disease, attacking persons advanced in life as well as the young, to whom the disease is almost exclusively confined when it is sporadic. The only case which we saw was that of a gentleman fifty-six years of age.

The case which we give to our readers was drawn up from memory, and is of course less complete than the author desired it should be; the anatomical details, however, are quite enough to prove the character of the affection. It is one of the peculiar features of the disease when epidemic, that the proportion of the severe and fatal cases is extremely great, and that the anomalous forms, such as the latent fever, and that terminating in perforation of the intestines, should become unusually frequent. We hope to present our readers with a history of the epidemic, as observed by the writer of the following letter, which will be found of much interest, especially to the numerous readers who reside in those parts of the country in which epidemics of this fever from time to time occur. In the present case the patient died of the local lesions, a very rare occurrence.]—*Eds. Ex.*

The patient was a coloured lad of about fifteen years old. He first made complaint of indisposition about 3 P. M. of the 13th March. Up to that time he had apparently enjoyed uninterrupted and robust health; had eaten his dinner on that day as usual with a good appetite, and passed the earlier hours of the afternoon in joyous sports with his playmates. His first complaints were of colicky pain in the abdomen, for which some domestic remedies were given him, without, however, affording any relief to his sufferings. On the contrary, his symptoms continued to grow more urgent until about 10 o'clock, P. M., when I was summoned to see him. On arrival I found him labouring under severe continued abdominal pain, which was much aggravated by pressure over the *epigastrium;* pressure upon other portions of the abdominal surface caused little or no increase of suffering; his tongue was uniformly coated with a slight *white* fur, as of milk smeared over the surface; pulse hard, and much accelerated in frequency. I began the treatment by drawing blood; gave castor oil and laudanum; directed a large sinapism to the abdomen, and his feet soaked in hot water; no relief following this treatment, after waiting an hour, I gave him a large dose of laudanum, and again drew blood, (the blood first drawn had already given the ordinary signs of the inflammatory condition.) This procured for him some relief, insomuch that he was enabled to sleep through the greater part of the night. It is perhaps worthy of remark that the easiest posture was either in the upright position, or upon the belly; called at an early hour in the morning, and found him without improvement; pulse much as on the previous evening; tongue somewhat more loaded; his bowels had not been moved; gave ten grains calomel, with directions to follow it with a tablespoonful of castor oil every two hours until freely purged. Called at 3 P. M.; still no better; bowels not yet moved; gave a purgative enema, which acted satisfactorily, and again drew blood; directed continuance of the oil, with laudanum, until freely purged. Called at 10 P. M.; evidently grows worse; bowels not yet acted on with sufficient freedom; repeated the enema, which brought away copious discharges of feculent stools, upon the surface of which the oil was seen floating; no relief following, had him placed in a warm bath, which for a time mitigated the severity of his sufferings; directed powders of calomel and ipecac. every two hours, and a blister over the whole abdomen; the pain had now shifted its seat to the left border of the umbilical region; wrote a note to a neighbouring physician, requesting a consultation at 8 o'clock next morning, and left him for the night under the apprehension of a troublesome case; upon arrival at the appointed hour, found that he had been dead an hour, being but forty hours from the first cognizance of any departure from health. Upon conferring together, the case appeared to us so unusual in its character, as to baffle every effort at a satisfactory diagnosis; we consequently requested and obtained permission to open the body.

*Autopsy four hours after death.*—Upon opening the abdomen we were much disappointed in finding every thing present the appearance of the most perfect health. No distention, no inflammation, nor any thing to arrest attention, except some slight and apparently very unimportant contractions of the colon. Upon opening the bowel at these points, all was found healthy. We examined several portions, always with the same result, the mucous coat of the stomach unchanged. We next separated a portion of bowel embracing the ileo-cœcal valve; upon opening this we found satisfactory signs of disease amply sufficient to explain the cause of death; the cœcal portion up to and embracing the valve, gave no signs of disease whatever, but immediately upon passing that septum they were seen in strong development; here were found a vast number of large pustular ulcerations, of an oval figure, the long diameter being longitudinal of the bowel, varying in size from a quarter of an inch to one and a half inches in length, by about the breadth of a finger nail; these covered a large part of the surface; the intermediate space was thickly studded with smaller ulcerations of the size of a grain of wheat, and differing somewhat from the larger ones; the whole, large and small, were covered by a thick adherent slough, of a dark gray colour, and raised above the surface of the bowel about the thickness of stout paste-board. We separated a portion of bowel of about thirty inches in length, and found the appearances to extend through the whole distance, although they became less numerous in proportion as we receded from the valve. These, so far as my memory serves me, are the principal facts of the case. The inference to my mind is irresistible, that the lesions we met with could not have run their whole course in the short time during which he complained of indisposition; yet it seems strange that such extensive alterations of structure involving organs so essential to health should have run on so near to the period of a fatal termination, and the general system show no signs of sympathetic irritation. This case has appeared to me particularly interesting from the belief that it may serve to throw light upon the obscurity which enveloped a severe and fatal epidemic which has prevailed to some extent through the surrounding neighbourhood during the past winter in the shape of nervous fever, of which I will give you some account on another occasion.

